# Supplementary material for: PDZD7-MYO7A complex identified in enriched stereocilia membranes
Source: eLife. 2016 Aug 15;5:e18312. doi: 10.7554/eLife.18312 (PMC5005036; doi:10.7554/eLife.18312)
Supplement: Reporting standards 2. — DOI: http://dx.doi.org/10.7554/eLife.18312.023 [file elife-18312-repstand2.doc]

**Reporting guidelines for mass spectrometry**

1. General features

1.1 Global descriptors

– Responsible person (or institutional role if more appropriate); provide name, affiliation and stable contact information: **Dr. Larry David, OHSU Proteomics Shared Resource, davidl@ohsu.edu, 503-418-1280**

– Instrument manufacturer and model: **Thermo Fisher Orbitrap Fusion ETD**

– Customizations (summary): **none**

2. Ion sources

*As each spectrum is acquired using only one ionization source, select the one that applies*

2.1 Electrospray Ionization (ESI)

– Supply type (static, or fed): **fed**

– Interface manufacturer, model: **Thermo Scientific Easy-Spray Nano ES**

– Sprayer type, manufacturer, model: **Thermo Scientific Easy-Spray column with integrated tip (P/N ES802 PepMap RSLC C18, 2um 100A)**

– Other parameters if discriminant for the experiment:

3. Post-source component

*As an MS spectrum or chromatogram performed on one instrument cannot be acquired using all existing analyzers and detectors, select the elements that apply*

3.1 Analyzer

– Ion optics, ‘simple’ quadrupole, hexapole, Paul trap, linear trap, magnetic sector, FT-ICR, Orbitrap: name of the analyzer(s): **Quadrupole for isolation of precursors ions, Orbitrap detection for MS1 and linear ion trap detection for MS2.**

3.2 Activation / dissociation

*The associated acquisition parameters are covered in 4.1*

– Instrument component where the activation / dissociation occurs: **ion-routing multipole**

– Gas type (when used) : **Helium**

– Activation / dissociation type : **HCD**

4. Spectrum and peak list generation and annotation

4.1 Data acquisition

– Software name and version: **Xcalibur version 3.0.63**

– Acquisition parameters: **Follows for each prep (experiment)**

**GIL1467 acquisition parameters**

Comments: MYO7a Chicken HB,Magnetic bead IP,1-D band,in-gel digest wProteaseMax,filter,dry,in 15ul of 5%FA.Load 15ul at 300 nl/min for 60min, partial loop inj,25cmcolumn,3.5 ul transport liquid.Pressure 380bar@2%B

Instrument Method: C:\Xcalibur\methods\PSR_methods\150min_dd_HCD_60min_direct_load.meth

Injection Volume (µl): 15.00

Orbitrap Fusion Method Summary

Creator: 3QKMFX1\Orbitrap Fusion Last Modified: 3/7/2014 5:37:59 PM by 3QKMFX1\Orbitrap Fusion

Global Settings

Method Duration (min)= 150

Source Type = eSourceType_NSI

Spray Voltage Pos = 2400

Spray Voltage Neg = 600

Sweep Gas Flow Rate Pos = 0

Sheath Gas Flow Rate Pos = 0

Aux Gas Flow Rate Pos = 0

Ion Transfer Tube Temp (C) = 275

Vaporizer Temp (C) = 0

Internal Cal Positive

m/z

445.12

Internal Cal Negative

m/z

Experiment 1

Start Time (min) = 60

End Time (min) = 140

Cycle Time (sec) = 5

Do data dependent experiment if no target species are found = False

Scan MasterScan

MSn Level = 1

Detector Type = Orbitrap

Orbitrap Resolution = 120K

Mass Range = Normal

Scan Range (m/z) = 350-1600

Maximum Injection Time (ms) = 50

AGC Target = 500000

Microscans = 1

S-Lens RF Level = 60

Use ETD Internal Calibration = False

DataType = Profile

Polarity = Positive

Source Fragmentation = False

Filter MIPS

Filter Type = MIPS

MIPS On = True

Filter DynamicExclusion

Filter Type = DynamicExclusion

Exclude after n times = 1

If occurs within (s) = 30

Perform dependent scan on single charge state per precursor only = False

Exclusion duration (s) = 60

Excl. Mass Width = ppm

Mass tolerance low = 0

Mass tolerance high = 0

Early expiration = False

Count = 1

S/N threshold = 2

Decision

Precursor Priority = MostIntense

Scan Event 1

IntensityGreaterThan: 5000

AND

ChargeRange: 2-6

Scan Event 1

Do data dependent experiment if no target species are found = False

Scan ddMSnScan

MSn Level = 2

Isolation Mode = Quadrupole

Top N= 0

Use Isolation m/z Offset = False

Isolation Window = 1.6

Multi-notch Isolation = False

Scan Range Mode = AutoNormal

FirstMass = 120

ActivationType = HCD

Collision Energy (%) = 30

Detector Type = IonTrap

Ion Trap Scan Rate = Rapid

Orbitrap Resolution = 30K

Scan Range (m/z) = 350-1000

Maximum Injection Time (ms) = 50

AGC Target = 10000

Microscans = 1

Use ETD Internal Calibration = False

DataType = Centroid

Polarity = Positive

Source Fragmentation = False

**GIL1649 20150216 95min acquisition parameters**

Comments: column 155147, trap 131647, dissolved in 22 ul of 5% formic acid and 1.2 ul injected for shotgun run

Instrument Method: C:\Xcalibur\methods\PSR_methods\New Methods\Gillespie_lab\95min_HCD_upickup_50msMS2_for_GIL1649_IP_MYO7_shotgun.meth

Injection Volume (µl): 1.20

Orbitrap Fusion Method Summary

Creator: 3QKMFX1\Orbitrap Fusion Last Modified: 2/16/2015 8:28:20 PM by 3QKMFX1\Orbitrap Fusion

Global Settings

Method Duration (min)= 95

Ion Source Type = NSI

Spray Voltage: Positive Ion (V) = 2400

Spray Voltage: Negative Ion (V) = 600

Sweep Gas (Arb) = 0

Ion Transfer Tube Temp (°C) = 275

APPI Lamp = Not in use

Internal Mass Calibration= User Defined Lock Mass

Pressure Mode = Standard

Default Charge State = 1

Internal Cal Positive

m/z

445.12003

Experiment 1

Start Time (min) = 5

End Time (min) = 90

Cycle Time (sec) = 3

Do data dependent experiment if no target species are found = False

Scan MasterScan

MSn Level = 1

Use Wide Quad Isolation = True

Detector Type = Orbitrap

Orbitrap Resolution = 120K

Mass Range = Normal

Scan Range (m/z) = 400-1500

Maximum Injection Time (ms) = 50

AGC Target = 200000

Microscans = 1

S-Lens RF Level = 60

Use ETD Internal Calibration = False

DataType = Profile

Polarity = Positive

Source Fragmentation = False

Filter MIPS

Filter Type = MIPS

MIPS On = True

Filter ChargeState

Filter Type = ChargeState

Include charge state(s) = 2-7

Include undetermined charge states = False

Include charge states 25 and higher = False

Filter DynamicExclusion

Filter Type = DynamicExclusion

Exclude after n times = 1

Perform dependent scan on single charge state per precursor only = False

If occurs within (s) = 30

Exclusion duration (s) = 30

Excl. Mass Width = ppm

Mass tolerance low = 10

Mass tolerance high = 10

Filter IntensityThreshold

Filter Type = IntensityThreshold

Signal Intensity = 5000

Decision

Precursor Priority = MostIntense

Scan Event 1

IntensityGreaterThan: 0

Scan Event 1

Do data dependent experiment if no target species are found = False

Scan ddMSnScan

MSn Level = 2

Top N= 0

Isolation Mode = Quadrupole

Isolation Window = 1.6

Use Isolation m/z Offset = False

Multi-notch Isolation = False

Scan Range Mode = Auto Normal

FirstMass = 110

ActivationType = HCD

Is Stepped Collision Energy On = False

Stepped Collision Energy (%) = 5

Multistage Activation = False

Neutral Loss Mass = 50.0001

Collision Energy (%) = 35

Is EThcD Active = False

Detector Type = IonTrap

Orbitrap Resolution = 30K

Ion Trap Scan Rate = Rapid

Maximum Injection Time (ms) = 50

AGC Target = 10000

Inject ions for all available parallelizable time = True

Microscans = 1

Activation Q = 0.25

Use ETD Internal Calibration = False

DataType = Centroid

Polarity = Positive

Source Fragmentation = False

**GIL1649 20150216 240min acquisition parameters**

Comments: column 155147, trap 131648, disolved in 22 ul of 5% formic acid and 4.8 ul injected for shotgun run (for IP samples, 6.4ul injected for totals)

Instrument Method: C:\Xcalibur\methods\PSR_methods\New Methods\Gillespie_lab\240min_HCD_upickup_50msMS2_for_GIL1649_IP_MYO7_shotgun.meth

Injection Volume (µl): 4.50

Orbitrap Fusion Method Summary

Creator: 3QKMFX1\Orbitrap Fusion Last Modified: 3/26/2015 11:55:56 AM by 3QKMFX1\Orbitrap Fusion

Global Settings

Method Duration (min)= 240

Ion Source Type = NSI

Spray Voltage: Positive Ion (V) = 2400

Spray Voltage: Negative Ion (V) = 600

Sweep Gas (Arb) = 0

Ion Transfer Tube Temp (°C) = 275

APPI Lamp = Not in use

Internal Mass Calibration= User Defined Lock Mass

Pressure Mode = Standard

Default Charge State = 1

Internal Cal Positive

m/z

445.12003

Experiment 1

Start Time (min) = 5

End Time (min) = 220

Cycle Time (sec) = 3

Do data dependent experiment if no target species are found = False

Scan MasterScan

MSn Level = 1

Use Wide Quad Isolation = True

Detector Type = Orbitrap

Orbitrap Resolution = 120K

Mass Range = Normal

Scan Range (m/z) = 400-1500

Maximum Injection Time (ms) = 50

AGC Target = 200000

Microscans = 1

S-Lens RF Level = 60

Use ETD Internal Calibration = False

DataType = Profile

Polarity = Positive

Source Fragmentation = False

Filter MIPS

Filter Type = MIPS

MIPS On = True

Filter ChargeState

Filter Type = ChargeState

Include charge state(s) = 2-7

Include undetermined charge states = False

Include charge states 25 and higher = False

Filter DynamicExclusion

Filter Type = DynamicExclusion

Exclude after n times = 1

Perform dependent scan on single charge state per precursor only = False

If occurs within (s) = 30

Exclusion duration (s) = 30

Excl. Mass Width = ppm

Mass tolerance low = 10

Mass tolerance high = 10

Filter IntensityThreshold

Filter Type = IntensityThreshold

Signal Intensity = 5000

Decision

Precursor Priority = MostIntense

Scan Event 1

IntensityGreaterThan: 0

Scan Event 1

Do data dependent experiment if no target species are found = False

Scan ddMSnScan

MSn Level = 2

Top N= 0

Isolation Mode = Quadrupole

Isolation Window = 1.6

Use Isolation m/z Offset = False

Multi-notch Isolation = False

Scan Range Mode = Auto Normal

FirstMass = 110

ActivationType = HCD

Is Stepped Collision Energy On = False

Stepped Collision Energy (%) = 5

Multistage Activation = False

Neutral Loss Mass = 50.0001

Collision Energy (%) = 35

Is EThcD Active = False

Detector Type = IonTrap

Orbitrap Resolution = 30K

Ion Trap Scan Rate = Rapid

Maximum Injection Time (ms) = 50

AGC Target = 10000

Inject ions for all available parallelizable time = True

Microscans = 1

Activation Q = 0.25

Use ETD Internal Calibration = False

DataType = Centroid

Polarity = Positive

Source Fragmentation = False

**GIL1651 240min acquisition parameters**

Comments: column 155147, trap 131648, elution from Myo7a IP, dissolved in 22 ul of 5% formic acid and 4.8 ul injected for shotgun run

Instrument Method: C:\Xcalibur\methods\PSR_methods\New Methods\Gillespie_lab\240min_HCD_upickup_50msMS2_for_GIL1649_IP_MYO7_shotgun.meth

Injection Volume (µl): 4.80

Orbitrap Fusion Method Summary

Creator: 3QKMFX1\Orbitrap Fusion Last Modified: 3/26/2015 11:55:56 AM by 3QKMFX1\Orbitrap Fusion

Global Settings

Method Duration (min)= 240

Ion Source Type = NSI

Spray Voltage: Positive Ion (V) = 2400

Spray Voltage: Negative Ion (V) = 600

Sweep Gas (Arb) = 0

Ion Transfer Tube Temp (°C) = 275

APPI Lamp = Not in use

Internal Mass Calibration= User Defined Lock Mass

Pressure Mode = Standard

Default Charge State = 1

Internal Cal Positive

m/z

445.12003

Experiment 1

Start Time (min) = 5

End Time (min) = 220

Cycle Time (sec) = 3

Do data dependent experiment if no target species are found = False

Scan MasterScan

MSn Level = 1

Use Wide Quad Isolation = True

Detector Type = Orbitrap

Orbitrap Resolution = 120K

Mass Range = Normal

Scan Range (m/z) = 400-1500

Maximum Injection Time (ms) = 50

AGC Target = 200000

Microscans = 1

S-Lens RF Level = 60

Use ETD Internal Calibration = False

DataType = Profile

Polarity = Positive

Source Fragmentation = False

Filter MIPS

Filter Type = MIPS

MIPS On = True

Filter ChargeState

Filter Type = ChargeState

Include charge state(s) = 2-7

Include undetermined charge states = False

Include charge states 25 and higher = False

Filter DynamicExclusion

Filter Type = DynamicExclusion

Exclude after n times = 1

Perform dependent scan on single charge state per precursor only = False

If occurs within (s) = 30

Exclusion duration (s) = 30

Excl. Mass Width = ppm

Mass tolerance low = 10

Mass tolerance high = 10

Filter IntensityThreshold

Filter Type = IntensityThreshold

Signal Intensity = 5000

Decision

Precursor Priority = MostIntense

Scan Event 1

IntensityGreaterThan: 0

Scan Event 1

Do data dependent experiment if no target species are found = False

Scan ddMSnScan

MSn Level = 2

Top N= 0

Isolation Mode = Quadrupole

Isolation Window = 1.6

Use Isolation m/z Offset = False

Multi-notch Isolation = False

Scan Range Mode = Auto Normal

FirstMass = 110

ActivationType = HCD

Is Stepped Collision Energy On = False

Stepped Collision Energy (%) = 5

Multistage Activation = False

Neutral Loss Mass = 50.0001

Collision Energy (%) = 35

Is EThcD Active = False

Detector Type = IonTrap

Orbitrap Resolution = 30K

Ion Trap Scan Rate = Rapid

Maximum Injection Time (ms) = 50

AGC Target = 10000

Inject ions for all available parallelizable time = True

Microscans = 1

Activation Q = 0.25

Use ETD Internal Calibration = False

DataType = Centroid

Polarity = Positive

Source Fragmentation = False

**Reporting guidelines for protein and peptide** **identification and characterization software**

1. General features

1.1 Global descriptors

– Date stamp (as YYYY-MM-DD) **n/a**

– Responsible person (or institutional role if more appropriate); provide name, affiliation and stable contact information **Peter Barr-Gillespie, Oregon Hearing Research Center, gillespp@ohsu.edu, 503-494-2936**

– Software name, version and manufacturer **MaxQuant 1.5.1.2**

– Customizations made to that software **n/a**

– Availability of that software **maxquant.org**

– Location of the files generated; parameter files, spectral data (input/output) **n/a**

2. Input data and parameters

2.1 Input data

– Description and type of MS data **Thermo .RAW**

– Availability of MS data (source of data, file format) **ProteomeXchange; .RAW files**

2.2 Input parameters

– Databases queried; description and versions (including number of entries searched **Gal_gal_v77.Galgal4.pep.all.fixed.extras.fasta (Ensembl chicken 77 with added PDZD7, CACNA2D2, ATP5B, CALB2, ESPN, PPIA, ACTG1, ACTB, ACTG2, ACTA1, ACTA2, and ACTC1 protein sequences)**

– Taxonomical restrictions applied **n/a**

– Description of tool and scoring scheme **MaxQuant 1.5.1.2**

– Specified cleavage agent(s) **Trypsin/P**

– Allowed number of missed cleavages **2**

– Additional parameters related to cleavage **n/a**

– Permissible amino acids modifications **Acetyl (protein N-term); oxidation (M)**

– Precursor-ion and fragment ion mass tolerance for tandem MS (when applicable) **n/a**

– Mass tolerance for PMF (when applicable) **n/a**

– Thresholds; minimum scores for peptides, proteins (probabilities, number of hits, other metrics) **n/a**

– Any other relevant parameters **n/a**

3. The output from the procedure

*The procedure might generate all or part of the elements described below (identified proteins, identified peptides, quantization information). Select the elements that apply.*

3.1 For identified proteins

– Accession code in the queried database **See data files**

– Protein description **See data files**

– Protein scores **See data files**

– Validation status **See data files**

– Number of different peptide sequences (without considering modifications) assigned to the protein **See data files**

– Percent peptide coverage of protein **See data files**

– Identity of supporting peptides **See data files**

– In the case of PMF, number of matched/unmatched peaks **n/a**

3.2 For identified peptides

– Sequence (indicate any deviation from the expected protein cleavage specificity) **See data files**

– Peptide scores **See data files**

– Chemical modifications (artefactual) and post-translational modifications (naturally occurring); sequence polymorphisms with experimental evidence (particularly for isobaric modifications) **See data files**

– Corresponding spectrum locus **n/a**

– Charge assumed for identification and a measurement of peptide mass error **See data files**

– Other additional information, when used for evaluation of confidence **See data files**

3.3 Quantitation for selected ions

– Quantitation approach (*e.g.* 4plex-iTRAQ, ICAT, cICAT, COFRADIC) **Relative iBAQ**

– Quantity measurement (*e.g.* integration of signals, use of signal intensity) **Integration of MS1 peak area**

– Data transformation and normalization technique (description of method and software) **MaxQuant 1.5.1.2 and Excel; see below**

– Number of replicates (biological and technical) **See below**

– Acceptance criteria (including measure of errors) **n/a**

– Estimates of uncertainty and the methods for the error analysis, including the treatment of relevant systematic error effects and the treatment of random error issues **n/a**

– Results from controls (when described) **n/a**

4. Interpretation and validation

– Assessment and confidence given to the identification and quantitation (description of methods, thresholds, values, etc,) **n/a**

– Results of statistical analysis or determination of false positive rate in case of large scale experiments **n/a**

– Inclusion/exclusion of the output of the software are provided (description of what part of the output has been kept, what part has been rejected) **All contaminants rejected; all reversed entries are present in output spreadsheet but are not used in quantitation calculations**

**Reporting guidelines for the peptide and protein quantification analysis.**

***1. General features***

– Experiment identifier or name **n/a**

– Responsible person or role **Peter Barr-Gillespie, Oregon Hearing Research Center, gillespp@ohsu.edu, 503-494-2936**

– Quantitative approach **Relative iBAQ; label-free extracted ion chromatograms, with intensity converted to iBAQ and iBAQ converted to riBAQ (Krey et al., J. Proteome Res. 13, 1034; 2013).**

***2. Experimental design and sample description***

2.1. Experimental design

– Groups **Three experimental samples:**

**Control IP elution—eluate from control beads after S7 passed over**

**MYO7A IP elution—eluate from 138-1 beads after control F/T passed over**

**Total—S7 fraction (RIPA solubilized enriched stereocilia membranes)**

– Biological and technical replicates **Immunoaffinity purification experiment was**

**conducted three times:**

**GIL1467—1 technical replicate each control and MYO7A**

**GIL1649—3 technical replicates each control and MYO7A;**

**3 Total replicates**

**GIL1651—2 technical replicates each control and MYO7A;**

**3 Total replicates**

2.2. Sample / Assay description

– Labeling protocol (if applicable) **n/a**

– Sample description **n/a**

Sample name **See table below**

Sample amount **See table below**

Sample labeling with assay definition, i.e. MS run / data set together with reporting ion mass, reagent or isotope labeled amino acid **n/a**

Replicates and/or groups **n/a**

Isotopic correction coefficients **n/a**

Internal references **n/a**

| **GIL1467** | **Injection volume (µl)** | **Total volume (µl)** | **Ears/ injection** | **Protein/ injection (µg)** |
| --- | --- | --- | --- | --- |
| Ctrl IP elution | 15 | 15 | 50 | unknown |
| Myo7a IP elution | 15 | 15 | 50 | unknown |
| Total | n/a | n/a | n/a | n/a |
|  |  |  |  |  |
| **GIL1649 (each replicate)** | **Injection volume (µl)** | **Total volume (µl)** | **Ears/ injection** | **Protein/ injection (µg)** |
| Ctrl IP elution | 4.5 | 22 | 97 | unknown |
| Myo7a IP elution | 4.5 | 22 | 97 | unknown |
| Total | 6.4 | 22 | 1 | 1.16 |
|  |  |  |  |  |
| **GIL1651 (each replicate)** | **Injection volume (µl)** | **Total volume (µl)** | **Ears/ injection** | **Protein/ injection (µg)** |
| Ctrl IP elution | 4.8 | 22 | 97 | unknown |
| Myo7a IP elution | 4.8 | 22 | 97 | unknown |
| Total | 6.4 | 22 | 1.31 | 1.16 |

***3. Input data***

*Description and reference of the dataset used for quantitative analysis (no actual values).*

– Input data type **Thermo .RAW**

– Input data format **n/a**

– Input data merging **Gel slice data for given replicate merged**

– Availability of the input data **ProteomeXchange**

***4. Protocol***

*Description of the software and methods applied in the quantitative analysis (including transformation functions, aggregation functions and statistical calculations).*

4.1. Quantification software name, version and manufacturer **iBAQ calculated with MaxQuant 1.5.1.2; all other calculations done with Microsoft Excel**

4.2. Description of the selection and/or matching method of features, together with the description of the method of the primary extracted quantification values determination for each feature and/or peptide **By MaxQuant**

4.3. Confidence filter of features or peptides prior to quantification **By MaxQuant**

4.4. Description of data calculation and transformation methods

– Missing values imputation and outliers removal **n/a**

– Quantification values calculation and / or ratio determination from the primary extracted quantification values **riBAQ for each protein or protein group is iBAQ divided by the sum of all non-contaminant, non-reversed iBAQ values**

– Replicate aggregation **Replicates averaged together**

– Normalization **riBAQ intrinsically normalizes**

– Inference protocol for calculating protein quantification values from peptide quantification values **By MaxQuant**

– Protocol specific corrections **n/a**

4.5. Description of methods for (statistical) estimation of correctness **Reversed database used for FDR estimate at protein level**

4.6. Calibration curves of standards **Assumption is that riBAQ is proportional to relative molar abundance (Krey et al., J. Proteome Res. 13, 1034; 2013)**

***5. Resulting data***

*The actual quantification values resulting from your quantification software together with their estimated confidence*

*5.1.* Quantification values at feature and/or at peptide level

– Primary extracted quantification values for each feature, with their statistical estimation of correctness **n/a**

– Quantification values for each peptide as a result of the aggregation of the values of the previous section (5.1.1), with their statistical estimation of correctness **Reversed database used for FDR estimate of 1% at peptide level**

5.2. Quantification values at protein level

– Basic / raw quantification values with statistical estimation of correctness **Reversed database used for FDR estimate of 1% at protein level**

– Transformed / aggregated / combined quantification values of the proteins at group level, with their statistical estimation of correctness **FDR re-evaluated after protein grouping**
